# A digitally supported multimodal lifestyle program to promote brain health among older adults (the LETHE randomized controlled feasibility trial): study design, progress, and first results

**DOI:** 10.1186/s13195-024-01615-4

**Published:** 2024-11-21

**Authors:** Anna Rosenberg, Helena Untersteiner, Anna Giulia Guazzarini, Markus Bödenler, Jeroen Bruinsma, Bianca Buchgraber-Schnalzer, Matteo Colombo, Rik Crutzen, Ana Diaz, Dimitrios I. Fotiadis, Hannes Hilberger, Simone Huber, Nico Kaartinen, Thomas Kassiotis, Miia Kivipelto, Jenni Lehtisalo, Vasileios S. Loukas, Jyrki Lötjönen, Mattia Pirani, Charlotta Thunborg, Sten Hanke, Francesca Mangialasche, Patrizia Mecocci, Elisabeth Stögmann, Tiia Ngandu, on behaf of the LETHE Consortium

**Affiliations:** 1 Department of Public Health, Finnish Institute for Health and Welfare, Helsinki, Finland; 2 Division of Clinical Geriatrics, Center for Alzheimer Research, Department of Neurobiology, Care Sciences and Society, Karolinska Institutet, Stockholm, Sweden; 3 Department of Neurology, Medical University of Vienna, Vienna, Austria; 4 Comprehensive Center for Clinical Neurosciences and Mental Health, Medical University of Vienna, Vienna, Austria; 5 Department of Medicine and Surgery, Section of Gerontology and Geriatrics, University of Perugia, Perugia, Italy; 6 eHealth Institute, FH JOANNEUM University of Applied Sciences, Graz, Austria; 7 Department of Health Promotion, Care and Public Health Research Institute, Maastricht University, Maastricht, the Netherlands; 8 Innovation2Grow (i2G), Milan, Italy; 9 Alzheimer Europe, Luxembourg, Luxembourg; 10 Unit of Medical Technology and Intelligent Information Systems, Department of Materials Science and Engineering, University of Ioannina, Ioannina, Greece; 11 Biomedical Research Institute, Foundation for Research and Technology - Hellas, FORTH-BRI, Ioannina, Greece; 12 GSRC, Division of Medical Physics and Biophysics, Medical University of Graz, Graz, Austria; 13 Kaasa Solution GmbH, Düsseldorf, Germany; 14 Computational BioMedicine Laboratory, Institute of Computer Science, Foundation for Research and Technology - Hellas, FORTH-ICS-CBML, Heraklion, Greece; 15 Theme Inflammation and Aging, Medical Unit Aging, Karolinska University Hospital, Solna, Sweden; 16 Ageing Epidemiology Research Unit, School of Public Health, Imperial College London, London, UK; 17 Institute of Public Health and Clinical Nutrition, University of Eastern Finland, Kuopio, Finland; 18 Combinostics Ltd, Tampere, Finland; 19 Department of Caring Sciences, Faculty of Health and Occupational Studies, University of Gävle, Gävle, Sweden

**Keywords:** Dementia, Cognitive decline, Prevention, Risk reduction, Randomized controlled trial, eHealth, mHealth, Technology

## Abstract

**Background:**

The Finnish Geriatric Intervention Study to Prevent Cognitive Impairment and Disability (FINGER) multimodal lifestyle intervention yielded cognitive and other health benefits in older adults at risk of cognitive decline. The two-year multinational randomized controlled LETHE trial evaluates the feasibility of a digitally supported, adapted FINGER intervention among at-risk older adults. Technology is used to complement in-person activities, streamline the intervention delivery, personalize recommendations, and collect digital biomarkers.

**Methods:**

Trial includes older adults (60–77 years) with digital readiness/experience with smart devices and increased dementia risk but without substantial cognitive impairment. Participants are enrolled at four sites (Austria, Finland, Italy, Sweden). At baseline, participants were randomized 1:1 ratio to 1) intervention i.e., structured multimodal lifestyle program (including diet, exercise, cognitive training, vascular/metabolic risk management, social stimulation, sleep/stress management) where in-person activities led by professionals are supported with an Android mobile phone application developed by the consortium (the LETHE App); or 2) control i.e., self-guided program (regular health advice; simplified App with no personalized/interactive content). All participants wear smartwatches to gather passive data (e.g., physical activity, sleep). Primary outcomes are retention, adherence, and change in validated dementia risk scores. Secondary outcomes include changes in lifestyle, cognition, stress, sleep, health-related quality of life, and health literacy. Additional outcomes (exploratory) include e.g. participant experiences and dementia-related biomarkers (Alzheimer’s disease blood markers, neuroimaging). A sub-study explores the feasibility of novel interactive technology (audio glasses, social robot).

**Results:**

Recruitment began in September 2022, and the last participant was randomized in June 2023. In total, 156 individuals were randomized (mean age 69 years, 65% women; balanced recruitment across the four sites). Vascular and lifestyle risk factors were common (e.g., 65% with hypertension, 69% with hypercholesterolemia, 39% physically inactive), indicating successful recruitment of a population with risk reduction potential. Trial will be completed by summer 2025. Retention until the first post-baseline visit at 6 months is high (*n* = 2 discontinued, retention 98.7%).

**Conclusion:**

LETHE provides crucial information about the feasibility of technology and a digitally supported FINGER lifestyle program to promote brain health. Digital tools specifically designed for older adults could offer potential for large-scale, cost-effective prevention programs.

**Trial registration:**

ClinicalTrials.gov (NCT05565170).

**Supplementary Information:**

The online version contains supplementary material available at 10.1186/s13195-024-01615-4.

## Background

Preventing late-life cognitive decline and dementia is a global health priority [1, 2]. Modifiable risk factors, many of which are related to lifestyle, cardiovascular health, and lack of cognitive/social stimulation, are estimated to account for at least 40% of dementia cases globally [3]. While there is obvious potential for individual- and population-level risk reduction, the etiology of late-life cognitive decline is complex and multifactorial, given the lifelong cumulative exposure to multiple risk and protective factors [4]. Thus, successful risk reduction approaches must be multifactorial, or multimodal, i.e., address several factors simultaneously [4, 5]. Such interventions can be non-pharmacological, pharmacological, or a combination of both [5]. In the landmark Finnish Geriatric Intervention Study to Prevent Cognitive Impairment and Disability (FINGER randomized controlled trial, RCT, ClinicalTrials.gov NCT01041989), a two-year multimodal lifestyle program (including dietary guidance, exercise, cognitive training, social activities, and vascular/metabolic risk management) showed clear positive effects on cognition, vascular health, and dementia risk estimates among 1260 community-dwelling older adults with dementia risk factors but no cognitive impairment [6–9]. The intervention was safe, feasible, and well-received by the participants [6, 10].

Integrating smart technology and digital tools is an attractive strategy to further optimize the FINGER model. Digitally supported interventions offer a flexible way to engage in the program and complete assessments e.g., at home. Digital tools could also help align the professional guidance with individual preferences, risk profile, and other needs to personalize the intervention. Increased flexibility and individualized adjustments support adherence, which can be a challenge in lifestyle interventions, particularly among individuals who have most room for improvement [11–15]. Importantly, passive data collection with smart devices could enable a closer monitoring of intervention effects and help capture subtle changes in risk factors and cognitive/functional status which are not detectable with standard assessments [16, 17].

As the adoption of digital services is growing among older adults, and the gap to younger adults is gradually narrowing, the window is now open for technology-assisted interventions to support brain health. In 2022, 64% of EU citizens aged 65–74 years used the Internet regularly (daily or at least once a week; 53% in 2019 and 42% in 2016), and of different activities, 36% mentioned searching for health information [18]. Digital health and lifestyle monitoring is also gaining popularity (eHealth, mHealth solutions). This underlines the need to develop feasible, effective, and scalable digital solutions for older adults, for cost-efficient risk factor self-management and dementia risk reduction.

The European LETHE project proposes a new multimodal precision prevention approach to address key challenges in dementia risk reduction with the help of technology [19, 20]. The multinational, two-year RCT presented in this paper investigates the feasibility of a digitally supported, adapted FINGER multimodal lifestyle intervention program among at-risk older adults. The primary objective of the RCT is 1) to assess participant retention, adherence, and engagement in the intervention which combines original FINGER in-person activities led by professionals with digital activities, and 2) to investigate change in dementia risk. Other objectives are to assess intervention-related changes in health, cognition, and risk factors, and to identify risk/protective factors and mechanisms behind aging and cognitive decline, also leveraging artificial intelligence and novel digital biomarkers.

## Methods

### Study design

LETHE (ClinicalTrials.gov NCT05565170) is a two-year pilot RCT conducted at four European sites: Medical University of Vienna (Austria), Finnish Institute for Health and Welfare (Finland), University of Perugia (Italy), and Karolinska Institutet (Sweden). At each site, eligible participants were randomized in a 1:1 ratio in blocks of four (centralized computer-based allocation performed by project partner FH Joanneum) to either a 1) structured digitally supported multimodal lifestyle program (= intervention; scheduled program designed and led by professionals) or 2) a self-guided multimodal lifestyle program (= control; regular health advice).

The structured LETHE intervention follows a hybrid approach where in-person intervention activities (on-site and remote; adopted from the original FINGER program) are supported by independent activities in the Android smartphone application designed by the LETHE Consortium (the LETHE App). The LETHE App includes personalized features and dynamic content supporting engagement in the lifestyle program and risk factor self-management. The self-guided group is encouraged to implement general health/lifestyle advice independently; this group has access to a simplified version (view) of the LETHE App including educational material selected by the study teams but no personalized content. All participants have the possibility to contact the study staff throughout the study, and they also receive support from so-called digital coaches (dedicated study staff members) who help with any technical questions and problems in person or by email/phone.

**Fig. 1:**
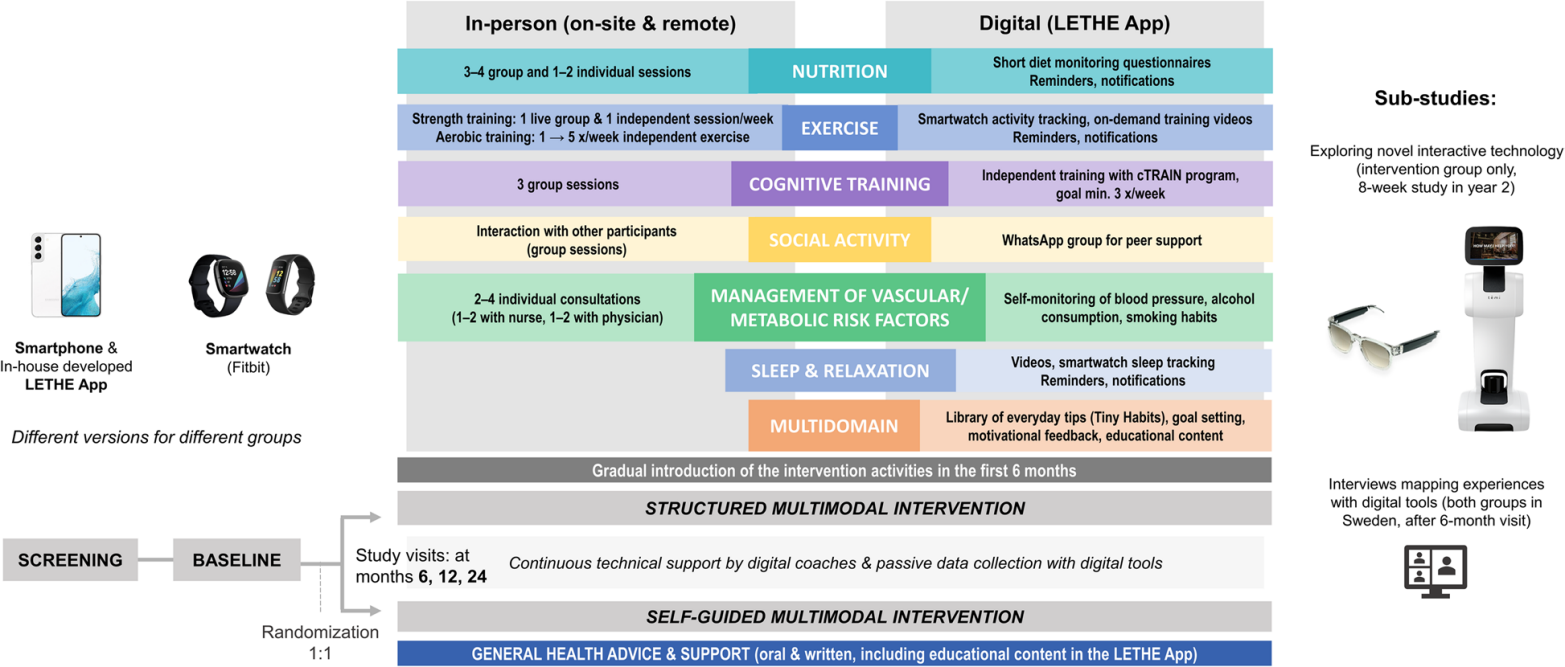
Overview of the LETHE trial design

Like in FINGER, blinding is pursued such that cognitive outcome evaluators are blinded to randomization and not involved in the intervention. Group allocation is not actively disclosed to participants. The main study visits, including clinical and cognitive assessments, take place at baseline and at 6, 12, and 24 months; additional data are collected digitally (actively via outcome-related questionnaires in the LETHE App and passively via monitoring through smart devices). During the second year, a subset of intervention group participants will be invited to join a two-month sub-study exploring novel interactive technology and its feasibility in the context of the LETHE intervention (audio smart glasses paired with a voice interaction app, a social robot). The overview of the study is presented in Fig. 1.

### Participants

Participants were recruited using a local social media (Facebook) advertisement in Sweden, Finland, and Austria. In Italy, local associations for older people were utilized, as well as printed posters which were distributed in the hospital/memory clinic. The study population in LETHE is similar as in FINGER: older adults (age 60–77 years) at risk of dementia based on the Cardiovascular Risk Factors, Aging, and Incidence of Dementia (CAIDE) Dementia risk score [21] and cognitive performance, but without any substantial cognitive impairment. LETHE participants were additionally required to have sufficient digital readiness to follow the intervention. The full inclusion and exclusion criteria are shown in Table 1.

**Table 1:** Participant inclusion and exclusion criteria

Inclusion criteria: • Age 60–77 years • Fluency in the local language (German, Italian, Finnish, or Swedish) • CAIDE Risk Score ≥ 6 • Cognition at mean level or slightly lower than expected for age, defined as having at least one of the following: MMSE ≤ 26, Consortium to Establish a registry for Alzheimer’s Disease (CERAD) word list learning ≤ 19/30, and/or CERAD word list delayed recall ≤ 75% • Sufficient digital skills to follow the intervention program and engage with the digital tools: Internet access, prior experience with smartphone, and an existing compatible smartphone or willingness to switch to a new smartphone provided by LETHE
Exclusion criteria: • Diagnosed or suspected dementia/substantial cognitive impairment (MMSE < 20); current/past use of Alzheimer’s disease/dementia medication • Significant neurologic disease e.g., Parkinson’s disease, Huntington’s disease, normal pressure hydrocephalus, brain tumor, progressive supranuclear palsy, seizure disorder, subdural hematoma, multiple sclerosis, or history of significant head trauma with persistent neurologic sequelae or known structural brain abnormalities • Diminished decision-making capacity, incapability to consent/complete study assessments, and any conditions preventing cooperation (based on clinical judgement) • Severe loss of vision, hearing, or communicative ability • Any conditions affecting safe intervention engagement e.g., malignancy, major depression, symptomatic cardiovascular disease, and revascularization within 1 year • Concomitant participation in an intervention trial (unless considered not to interfere with LETHE)

### Study procedures and data collection

During recruitment, individuals expressing their interest to join the study were first pre-screened over the phone to check basic inclusion criteria to reduce screening failures (age, digital readiness/skills, and CAIDE score). Digital readiness was assessed by interviewing the participant about their prior use of digital devices (which device, how often and for which purposes, and if the participant is comfortable with using Internet and email (e.g., search for information online, read and send emails)), about their current internet access, and willingness to use a phone that is compatible with the LETHE App. Potentially eligible persons were invited to full screening for detailed eligibility assessment, including cognitive testing and interview to confirm absence of medical exclusion criteria (Table 1). Screening was conducted on-site (Austria, Italy, Sweden) or online (Finland).

At baseline, in addition to conducting clinical, cognitive, and other assessments, the study staff installed the LETHE App on participants’ smartphones (a new smartphone was provided if participant’s existing phone was not compatible with the technical requirements of LETHE). Participants were requested to complete a set of questionnaires (e.g., lifestyle-related) in the App within two weeks from the baseline visit. Once completed, participants were randomized, and the full version of the App was activated (different view and content for the intervention and control groups). Successively, an extra visit with the digital coach was arranged to introduce the entire App content to the participants, and to hand out a smartwatch (intervention group: Fitbit Sense; control group: Fitbit Charge 5) and a tablet computer for those intervention group participants who did not yet own one.

After baseline, the main study visits take place at 6, 12, and 24 months, to collect clinical and cognitive outcome data. At these timepoints, participants in both groups also complete outcome-related questionnaires in the LETHE App. Other digital data collection takes place continuously throughout the trial, i.e., passive monitoring through the phone, LETHE App, and smartwatch. Table 2 summarizes the data collected at different timepoints. The trial also includes biomarker studies: at baseline and end of study, blood samples are collected for e.g. analysis of Alzheimer’s disease (AD) blood markers and MRI is conducted (in a subsample) across all sites.

**Table 2:** Overview of assessments conducted for all participants

	**Screening**	**Baseline**	**Month 6**	**Month 12**	**Month 24**
*General participant characteristics*					
Demographics (age, sex), education, marital & socioeconomic status, living situation	x	x			x
*Self-reported health status & medical history*					
Medical history, diagnoses, medication use		x	x	x	x
Family history of dementia, CVD, diabetes		x		x	x
*Measurements*					
Height & weight (BMI), blood pressure, hip-waist circumference	x	x	x	x	x
Routine blood tests^a^		x	x	x	x
DNA sample (for *APOE* genotyping)		x			
Blood samples for research purposes^b^		x			x
Brain MRI		x			x
*Cognition & function*					
CERAD word list learning & delayed recall	x				
MMSE	x			x	x
Subjective cognitive concerns		x	x	x	x
CDR, CDR-SB		x			x
Extended NTB		x		x	x
Digital cognitive assessment battery (cCOG)^c^		x		x	x
IADL		x		x	x
*Physical performance*					
SPPB, grip strength, timed 10-m dual task test		x			x
*Lifestyle questionnaires*^*d*^					
Food and eating habits		x		x	x
Physical activity		x		x	x
Smoking & alcohol consumption		x		x	x
Cognitive & social activities		x		x	x
Sleep problems (ISI)		x		x	x
*Mood, depressive symptoms, quality of life*^*d*^					
Stress-related symptoms (PSS-14)		x		x	x
Depressive symptoms (CES-D)		x		x	x
Zung depression scale^e^		x			
Health-related quality of life (RAND-36)		x		x	x
*Health literacy, attitudes & experiences*^*d*^					
Health literacy (HLS-EU-Q16)		x			x
Attitudes to dementia prevention and risk reduction (selected items of MCLHB-DRR scale)		x			x
Digital skills, Internet use, smartphone habits		x			
Assessment of experiences with the LETHE App (SUS)		x	x		x
Reasons for participation		x			
Feedback on participation				x	x
*Passive data collection through the smartwatch, smartphone & LETHE App*					
Activity log, sleep parameters, heart rate, heart rate variability, step count, SpO2 (smartwatch)	*Continuous data collection throughout the trial*				
Phone sensor & app data (count of nearby Bluetooth devices, phone usage data, data on LETHE App usage)					
Data from the cTRAIN cognitive training program (e.g., logins, performance)^f^					

^a^Total cholesterol, HDL, LDL, triglycerides, HbA1c, CRP, fasting glucose, creatinine, ASAT, ALAT, GGT

^b^Analysis of aging- and dementia-related biomarkers e.g., AD markers (p-tau181, NfL)

^c^Using personal computer/tablet or a device at the study center, approx. 1 month after each NTB

^d^Questionnaires completed independently in the LETHE App

^e^Assessed in Austria, Finland, and Italy

^f^Only intervention group

*Abbreviations*: *ADL* activities of daily living, *APOE* apolipoprotein E, *ALAT* alanine aminotransferase, *ASAT* aspartate aminotransferase, *BMI* body mass index, *CDR* Clinical Dementia Rating, *CDR-SB* Clinical Dementia Rating Sum of Boxes, *CERAD* Consortium to Establish a Registry for Alzheimer's Disease, *CES-D* Center for Epidemiologic Studies Depression Scale, *CRP* C-reactive protein, *CVD* cardiovascular disease, *GGT* gamma-glutamyl transferase, *HDL* high-density lipoprotein, *HLS-EU-Q16* 16-item European Health Literacy Survey Questionnaire, *IADL* Instrumental Activities of Daily Living, *ISI* Insomnia Severity Index, *LDL* low-density lipoprotein, *MCLHB-DRR* The Motivation to Change Lifestyle and Health Behaviours for Dementia Risk Reduction scale (Kim et al., Dementia and Geriatric Cognitive Disorders Extra, 2014), *MMSE* Mini-Mental State Examination, *MRI* magnetic resonance imaging, *NfL* neurofilament light chain, *NTB* Neuropsychological Test Battery, *PSS-14* 14-item Perceived Stress Scale, *p-tau181* phosphorylated tau 181, *SPPB* Short Physical Performance Battery, *SUS* 10-item System Usability Scale

### Outcome assessments

#### Primary outcomes

The primary outcomes are: 1) feasibility of the digitally supported multimodal lifestyle intervention and 2) change in dementia risk based on validated risk scores.

*Feasibility* is assessed based on retention rate (proportion of randomized participants completing the trial in each group) and adherence to the intervention. A retention of 65% (max. 35% drop-out) is considered successful. Reasons for discontinuation are recorded. In terms of adherence, both digital and non-digital intervention activities will be considered. The following will be assessed: usage of and engagement with the LETHE App and smartwatch (e.g., frequency and duration of logins, wear time), and participation in study visits and intervention-related activities/meetings. Feasibility is further explored by investigating participants’ opinions on the intervention and the usability of the LETHE App / digital tools (quantitative assessment with the System Usability Scale [22] and qualitative interviews in a subsample).

*Change in dementia risk* is assessed based on two scales, CAIDE [21] and LIfestyle for BRAin health (LIBRA) index [23]. Both are validated tools to estimate risk of late-life dementia [24, 25], and findings from FINGER suggest that they can be useful in quantifying risk reduction and prevention potential in an at-risk population and trial context [8, 9]. CAIDE considers non-modifiable and modifiable risk factors: age, education, sex, systolic blood pressure (BP), body mass index (BMI), total cholesterol, and physical activity. Total score is the sum of the points assigned for each factor (range 0–15; higher scores indicate higher risk). LIBRA consists of a weighted sum score of modifiable risk and protective factors; factors in the original version include coronary heart disease, diabetes, hypercholesterolemia, hypertension, depression, obesity, smoking, physical activity, renal disease, alcohol use, cognitive activity, and diet (theoretical range from –5.9 to + 12.7; higher scores indicate higher risk).

#### Secondary outcomes

Secondary outcomes include changes in lifestyle/adherence to healthy lifestyle, stress-related symptoms, sleep problems, health-related quality of life, health literacy, and cognition (composite z-score of 14 tests in the extended Neuropsychological Test Battery, NTB [26], adapted from the FINGER [6] and MET-FINGER [27] RCTs). The assessment methods and scales are summarized in Table 3.

**Table 3:** Methods and scales to assess secondary outcomes

**• Lifestyle/adherence to healthy lifestyle**: Composite score developed in FINGER, based on self-reported data on exercise, diet, vascular factors, and cognitive/social activity (Barbera et al., Alzheimer’s & Dementia, 2021. doi:https://doi.org/10.1002/alz.053388) **• Stress-related symptoms**: Perceived Stress Scale-14 (Cohen et al., Journal of Health and Social Behavior, 1983. doi:https://doi.org/10.1002/alz.05338810.2307/2136404) **• Sleep problems**: Insomnia Severity Index (Morin, Insomnia: Psychological assessment and management, 1993) **• Health-related quality of life**: RAND-36 scale (Hays et al., Health Econ, 1993. doi:https://doi.org/10.1002/hec.4730020305) **• Health literacy**: 16-item European Health Literacy Survey Questionnaire (Sørensen et al., Eur J Public Health, 2015. doi: https://doi.org/10.1093/eurpub/ckv043) **• Cognitive performance**: Composite z-score of the following 14 NTB tests: WMS-III, WMS-R, WMS-IV Logical Memory, immediate; Logical Memory, delayed; WMS-R Visual Paired Associates, immediate; Visual Paired Associates, delayed; Digit Span; RAVLT learning; RAVLT delayed recall; CERAD category fluency; Category fluency fruits and vegetables; TMT A; TMT B (shifting score B–A); Shortened 40-stimulus Stroop Test condition 2; Stroop test condition 3 (interference score 3–2); WAIS-IV Digit Symbol Substitution Test	

*Abbreviations*: *CERAD* Consortium to Establish a Registry for Alzheimer's Disease, *FINGER* Finnish Geriatric Intervention Study to Prevent Cognitive Impairment and Disability, *NTB* Neuropsychological Test Battery, *RAVLT* Rey Auditory Verbal Learning Test, *TMT* Trail Making Test, *WAIS* Wechsler Adult Intelligence Scale, *WMS* Wechsler Memory Scale

#### Exploratory/other outcomes

Exploratory outcomes include changes in individual lifestyle domains and risk factors, mood/depressive symptoms, sleep quality/duration, physical performance, and cognitive and functional measures. Changes in AD/dementia-related biomarkers will also be explored. The assessment methods and scales are summarized in Table 4.

**Table 4:** Methods and scales to assess exploratory/other outcomes

• **Physical activity**: objectively measured with the smartwatch and self-reported based on an adapted Minnesota questionnaire (Taylor et al., J Chronic Dis, 1978. doi: https://doi.org/10.1016/0021-9681(78)90058-9) • **Nutrient and food intake**: questionnaire adapted from the versions used e.g., in previous multimodal prevention trials (FINGER, MIND-AD_mini_, HATICE) • **Vascular/metabolic factors**: BP, BMI, waist circumference, blood lipids, fasting glucose, HbA1c, smartwatch data on heart rate / heart rate variability • **Mood/depressive symptoms**: Center for Epidemiologic Studies Depression Scale (Lewinsohn et al., Psychol Aging, 1997. doi: https://doi.org/10.1037//0882-7974.12.2.277); count of nearby Bluetooth devices via passive phone sensors as a proxy for social interaction • **Sleep**: smartwatch data on quality, duration, fragmentation parameters • **Physical performance**: grip strength, timed 10-m dual task test, Short Physical Performance Battery (Guralnik et al., J Gerontol, 1994. https://doi.org/10.1093/geronj/49.2.m85) • **Cognition, function**: Individual NTB domains (memory, executive function, processing speed); CDR-SB (Morris, Neurology, 1993. https://doi.org/10.1212/wnl.43.11.2412-a); IADL (Lawton & Brody, Gerontologist,1969. https://doi.org/10.1093/geront/9.3_Part_1.179); and digital cognitive test battery cCOG (Rhodius-Meester et al., Alzheimers Dement (Amst), 2020. https://doi.org/10.1002/dad2.12083) • **AD/dementia fluid biomarkers**: blood NfL and p-tau181 (Simoa) • **AD/dementia imaging biomarkers:** computationally assessed MRI volumetry, white matter lesions, and ratings for MTA, GCA, and Fazekas (Koikkalainen et al., Neuroimage Clin, 2016. https://doi.org/10.1016/j.nicl.2016.02.019; Koikkalainen et al., Eur Radiol, 2019. https://doi.org/10.1007/s00330-019-06067-1)	

*Abbreviations*: *BMI* body mass index, *BP* blood pressure, *CDR-SB* Clinical Dementia Rating Sum of Boxes, *FINGER* Finnish Geriatric Intervention Study to Prevent Cognitive Impairment and Disability, *GCA* global cortical atrophy, *HATICE* Healthy Aging Through Internet Counselling in the Elderly, *IADL* Instrumental Activities of Daily Living, *MIND-AD*_*mini*_ Multimodal Preventive Trial for Alzheimer’s Disease, *MRI* magnetic resonance imaging, *MTA* medial temporal lobe atrophy, *NfL* neurofilament light chain, *NTB* Neuropsychological Test Battery, *p-tau181* phosphorylated tau 181

### Intervention

#### Structured multimodal lifestyle program (intervention group)

The LETHE intervention is a digitally supported, structured multimodal lifestyle program delivered partly in-person and partly digitally using the LETHE App and other digital tools (tablet, smartwatch). The intervention domains are 1) Dietary guidance, 2) Physical activity, 3) Cognitive training, 4) Monitoring and management of vascular and metabolic risk factors, 5) Social stimulation, and 6) Sleep and stress management (only digital). The in-person intervention activities are organized both face-to-face and remotely as online meetings. The activities are aligned with the original FINGER and the more recent FINGER-based RCTs [27, 28].

The design and development of the multimodal lifestyle intervention program are centrally coordinated and harmonized. The intervention activities at the four sites share common key principles and similar schedules to ensure comparable intervention content and intensity. Detailed staff manuals guarantee harmonized intervention delivery across all study sites. Certain local adaptations, as well as tailoring to individual participant needs are nevertheless allowed to optimize feasibility and efficacy. Also, each site designs the detailed intervention delivery (e.g., balance between face-to-face and online meetings) depending on local arrangements and feasibility, as well as participant preferences.

To support adherence, the different intervention domains were introduced gradually during the first six months of the trial, starting with individual vascular risk factor and dietary consultations which were followed by cognitive and finally exercise group sessions. Participants gained access to all the intervention content in the LETHE App directly after randomization and the digital introduction visit.

#### Dietary guidance

The dietary intervention aligns closely with the principles of FINGER [29] and is based on general dietary recommendations with special attention on nutritional issues common among older adults. Local adjustments are allowed to align with national dietary recommendations. The key dietary intervention goals in nutrient and food intake levels are summarized in Supplementary Table 1 (Additional file 1). The intervention is delivered through individual consultations (1–2 sessions, approx. 30–45 min each) and small group sessions (3–4 sessions, approx. 45–60 min each) with a trained nutritionist. Individual consultations include a thorough assessment of the participant’s current dietary habits and provide tailored, practical advice on how to improve diet and implement changes in daily routines. All recommendations are adjusted according to individual needs considering e.g., health status and weight. Group sessions provide more information and support the implementation of relevant dietary changes. Group support is exploited through joint discussions. Participants are encouraged to invite their spouse/partner to join the sessions.

#### Physical activity

The physical activity intervention is based on FINGER and the international guidelines for older adults [30]. The program combines aerobic, strength, and balance training, with progressively increasing intensity and frequency. It is tailored to meet individual needs, e.g., fitness level, health status, and personal preferences. The goal is to make permanent changes to include physical activity into everyday life. Participants are encouraged to lead a more active and less sedentary life and provided with practical advice on how to incorporate activity in their daily routines.

The exercise program is led by a physiotherapist or trained professional experienced in working with older adults, and consists of the following components: an initial motivational consultation and muscle strength testing to define the optimal training load; group sessions on strength training (at least one weekly 30–60 min session at the gym and/or online); and independent aerobic exercise (planned together with the professional based on participants’ needs and preferences). The progression pattern for the training is shown in Supplementary Table 2 (Additional file 1). Participants are advised to use the on-demand video material in the LETHE App to reach the goal of two weekly strength training sessions. To track their activities and self-monitor exertion level to ensure right intensity level, participants are encouraged to utilize their smartwatches.

Group sessions on strength training follow a similar structure and concept regardless of whether they take place on-site or online (e.g., duration, targeted muscle groups). Remote sessions rely on bodyweight exercises, resistance bands, and exercises utilizing everyday tools at home (e.g., chairs, bottles). Even if most group sessions were remote, all sites organize a few on-site sessions at the beginning and end of the intervention, as well as in-between, to ensure safety and proper exercise techniques, and to facilitate grouping, support motivation, and monitor progression.

#### Cognitive training

The cognitive intervention is delivered through group sessions (3 sessions, approx. 60 min each) with a psychologist or other trained professional, and independent cognitive training with a web application available via the LETHE App (cTRAIN, provided by Combinostics Ltd and adapted from the program used in FINGER [31]). The group session topics are cognitive abilities, aging-related changes in cognition, brain plasticity, factors affecting cognition (e.g., sleep, stress, mood), and tips to stay mentally fit. The first group session is dedicated to introducing the cTRAIN program; after that the participants have access to the program for the whole trial duration. cTRAIN includes six games to train working memory (visuo-spatial span task), executive functions (two working memory updating tasks, spatial and verbal), mental speed (set-shifting task), and episodic memory (word associate task i.e., word triplets and a classic memory-game). The software introduces automatically different tasks in a sequential order (two games available at a time for each two-week period). With improving performance, the difficulty of the games increases, and more advanced levels are unlocked. Participants are recommended to train three times per week for 10–15 min/session, but they can train more if they wish. Activity and performance are registered automatically, and participants can monitor selected statistics (completed sessions, high scores).

#### Monitoring and management of vascular/metabolic risk factors

Risk factor monitoring and management is based on country-specific evidence-based guidelines. The goal is to identify risk factors and motivate participants to take action by giving tailored, targeted counselling and personalized feedback taking into account individual situation and motivation. Risk factors/conditions to cover include BP/hypertension, dyslipidemia, diabetes/ glucose levels, smoking, alcohol consumption, weight management (acknowledging the complex role of weight changes in old age and risk of frailty/malnutrition), and other relevant lifestyle factors. The intervention includes 1–2 individual consultations with a nurse and physician, respectively (in total 2–4 sessions, approx. 45–60 min each). Extra measurements of e.g., BP or waist circumference can be conducted to support the consultation. If medication initiation or adjustment is needed, the study physician writes a prescription, or the participant is recommended to contact (or referred to) the regular health care provider.

#### Social stimulation

In previous multimodal prevention RCTs, social engagement was crucial to support motivation and intervention adherence [10, 32]. In LETHE, social activities are therefore strongly supported and stimulated through regular group meetings. Group sessions are designed to facilitate open discussions and social interaction among participants. This interaction is also encouraged outside the scheduled sessions, e.g., via local WhatsApp group chats.

#### Sleep and stress management

Stress and sleep are acknowledged as relevant factors for brain health, although more evidence is needed of their potential causal role as risk factors [33, 34]. Stress and sleep problems can nevertheless interfere with health as a whole and prevent from making sustained lifestyle changes. No separate in-person activities are organized in this domain, but the topics are addressed as part of other consultations as applicable. Participants can independently monitor sleep with the smartwatch and access relaxation videos in the LETHE App.

#### Self-guided multimodal lifestyle program (control group)

Participants in the control group are advised to build their own healthy lifestyle program based on the standard advice they receive at the main study visits and through the simplified LETHE App. Participants are encouraged to independently implement relevant changes in their daily routine. The simplified LETHE App includes a library of educational material selected by the local study teams, but none of the personalized features or notifications (see next section and Fig. 1). The smartwatch worn by the control group is a simpler model than the one worn by the intervention group (serves mostly passive data collection, yet allows e.g. physical activity tracking). Like the intervention group, the control group receives the results of routine blood tests (e.g., lipids), information about their meaning, and if needed, advice to seek medical care.

#### LETHE App

**Fig. 2:**
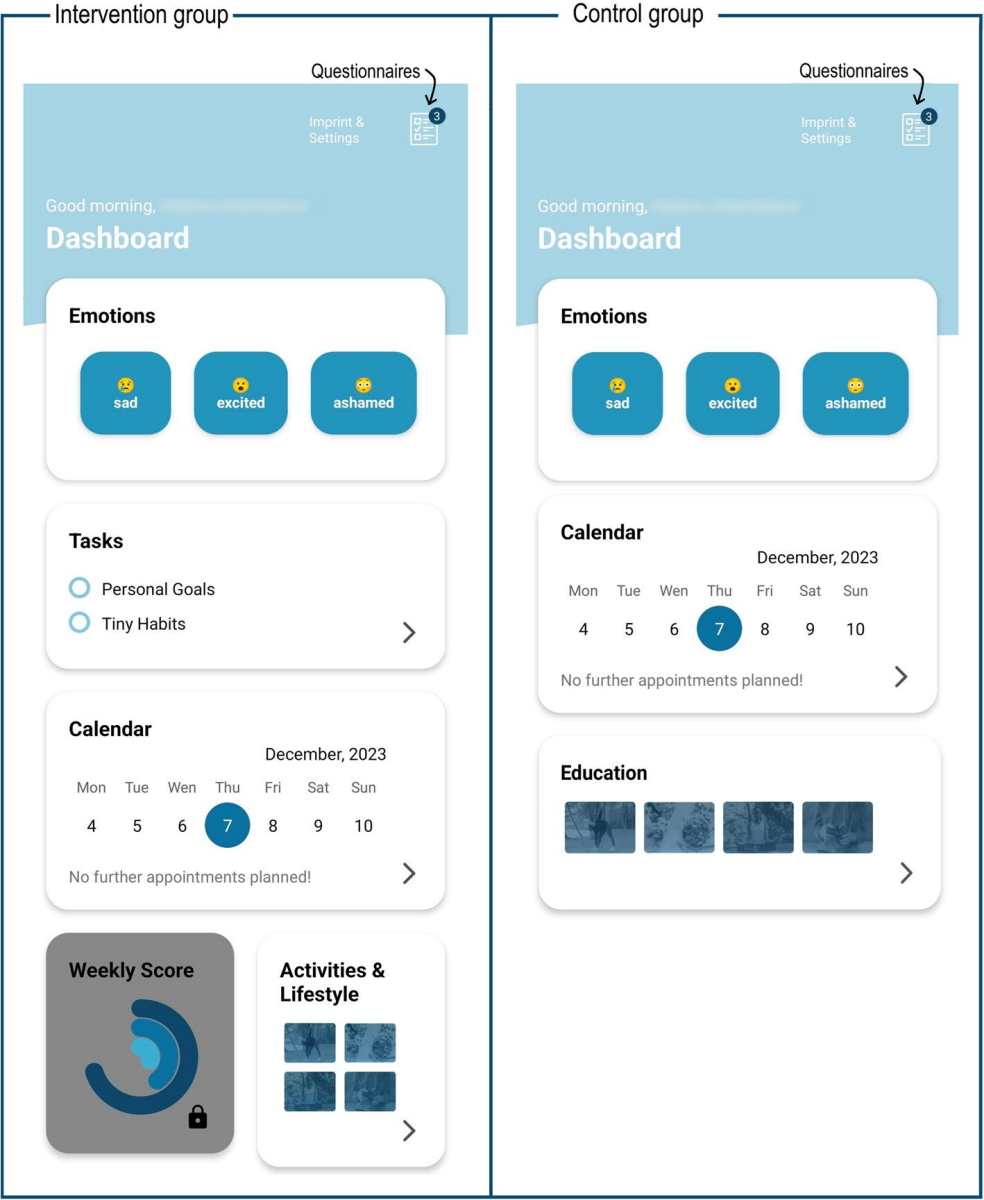
Overview (front page) of the different LETHE App views (full view for the intervention group, simplified view for the control group)

Given the lack of existing apps that are appropriate for the LETHE target group and aligned with the principles of the FINGER intervention model, a new app was in the project to support the intervention delivery and trial data collection. The LETHE App is an Android native app; the development process and technical implementation are described elsewhere (Hilberger et al., in preparation). In brief, the LETHE App features two different views (full view with all functionalities for the intervention group; simplified view with selected functionalities for the control group). Figure 2 shows the two different overviews (dashboards) of the LETHE App. All content is available in English and the four trial languages (Finnish, German, Italian, Swedish). Translations were provided and checked by the local study teams.

The key features of the LETHE App are summarized in Fig. 1. Features for both study groups include: a calendar (to view and keep track of study appointments and join online meetings if applicable), questionnaires (for outcome-related assessments at timepoints shown in Table 2), option to record current mood (by selecting emoticons), and a library of educational content covering all domains of the lifestyle program (external websites). This material was selected by the study teams to ensure it is appropriate for this target group, evidence-based, reliable, and aligned with the principles of the lifestyle intervention as well as local guidelines. The full App includes several additional features designed to support the delivery of the multimodal lifestyle intervention and encourage independent engagement in healthy activities. Some of the features and activities can be viewed and modified by the study staff on a web portal (LETHE Clinical Trial Management System, CTMS) which helps deliver the intervention in a more personalized way.

For diet, a short questionnaire is available in the App for simple self-monitoring of healthy diet adherence (e.g., consumption of fish, processed meat, and fruit and vegetables). The questionnaire is divided into three blocks, each of which appears weekly (Additional file 1, Supplementary Table 3). For physical activity, on-demand exercise videos focusing on strength and balance training are available (selected or filmed by the local study teams to accommodate different fitness levels). These videos complement the in-person group sessions. To relax or help fall asleep, YouTube videos with soothing melodies and breathing exercises are available (selected by the study teams). For cognitive training, participants have access to the cTRAIN program through the LETHE App. cTRAIN, as well as all videos in the LETHE App, can also be opened on a tablet or computer, by scanning a QR code or sending the link by e-mail. For social stimulation, participants are offered the possibility to connect with each other in a local WhatsApp group chat. For vascular risk factor control, the App includes a diary for self-monitoring of smoking, alcohol consumption, and BP. The professionals can view the entries in the CTMS, and results can be discussed at the individual consultations.

The full LETHE App also includes a feature for personal goals. The goals are discussed and set together with the professional to ensure they are relevant and follow the SMART principles (specific, measurable, achievable, realistic, time-bound). Goals are displayed in the App as items on a to-do list, and they can be ticked off once reached. The full App also includes a library of practical everyday tips and behavioral suggestions to help individuals implement healthy habits into the daily routine (so called tiny habits, based on habit formation theory [35, 36]). The library was originally created for a large Finnish type 2 diabetes online prevention RCT [37] (library publicly available in Finnish under CC BY 4.0 license [38]). Lifestyle domains covered were diet, exercise and sedentary lifestyle, mental wellbeing, sleep, smoking, and social interaction. For LETHE, the library was reviewed, translated, and partly adapted and expanded by the study teams to ensure appropriateness for our context. For example, the dietary and exercise advice was aligned with the principles of FINGER, and a new section was developed for cognitive stimulation. Participants can choose new habits and unselect old ones as often as they wish (total number of available habits > 500; examples in Additional file 1, Supplementary Table 4). Participants can indicate regularly if the habits were successfully implemented or not. Finally, a feature for personalized semi-automated feedback is currently under development. Based on data from the smart devices, motivational feedback messages will be sent, and a weekly performance/adherence score will be calculated and shown in the App (Fig. 2).

#### Exploring novel interactive technology (sub-study)

A subset of intervention group participants will be invited to test novel interactive technology as a complementary way to engage with the LETHE App. Two different technologies are tested: a social robot (Temi) and audio smart glasses (FAUNA Spiro) (Fig. 1). The robot can navigate around the house and display LETHE App content on a touch display. It can also assist by reminding about intervention-related tasks. The audio glasses have a Bluetooth microphone and headphones. In combination with a voice interaction app, glasses enable hands-free interaction with the LETHE App functionalities. Instead of reading on the screen and typing answers to questions as done with the smartphone, participants can listen to App contents and enter information through voice interaction. Both devices are CE-certified.

### Statistical considerations

LETHE is an RCT assessing primarily feasibility. In line with CONSORT extension guidelines for such RCTs [39], no formal sample size calculations were conducted. Sample size is in line with other feasibility RCTs testing multimodal preventive lifestyle interventions in similar populations [28, 40–42], and many ongoing studies assessing digital brain health interventions [43]. The two study groups will be compared to assess differences in the trial outcomes, as applicable.

### Ethical and safety aspects

Trial was approved by local ethical committees in all four countries. All participants provided a written informed consent prior to enrollment. Separate consents were obtained for screening and the full trial; an additional consent will be obtained for the sub-study. A verbal informed consent was allowed for remote screening. Participants’ study partners/informants provided their own consent (involving a partner e.g., family member is recommended but not mandatory).

The multimodal lifestyle intervention is not expected to cause harm or involve major health risks [6]. No interim safety analysis is therefore planned. Information about adverse events is collected at the study visits and with a brief digital questionnaire every three months. All participants are informed about the results of their routine health examinations, blood tests, and any other relevant findings concerning their health. If needed, participants are either referred to medical care or advised to contact their regular health care provider. Participants are covered by local insurance.

### Data protection and privacy

All study documentation collected at the sites is stored securely in local premises, labeled with a unique participant ID, and kept apart from identifying information. All data collected via the LETHE App, CTMS (including electronic case report forms), smartwatch, and phone sensors are stored on a secure cloud-based server with restricted access (hosted by EGI Foundation in the Netherlands). In addition to the participant ID, participants have unique dummy Google accounts to login the LETHE App and the other web applications used in the RCT (e.g., cognitive training program). Dummy accounts enable the linkage of data from different sources on the server. After the end of the LETHE project, data are transferred to the study sites and stored locally. Third parties e.g., Fitbit, collect and process certain data for their own purposes via their products (e.g., usage of smartwatch and related app); this is explained to the participants in the informed consent form. Third parties do not have access to the trial data.

### Public involvement

An Advisory Board (AB) including members of the public with an interest in or affected by cognitive impairment was set up at the beginning of the project (work led by Alzheimer Europe in collaboration with other partners). The AB consists of seven members from Austria, Finland, Italy, and Sweden. The AB has provided feedback on the trial design and intervention concept, participant materials, digital devices, and issues concerning recruitment, ethical aspects, and data protection. The AB contribution is described in detail elsewhere (Rosenberg et al., in preparation).

## Study progress and first results

**Fig. 3:**
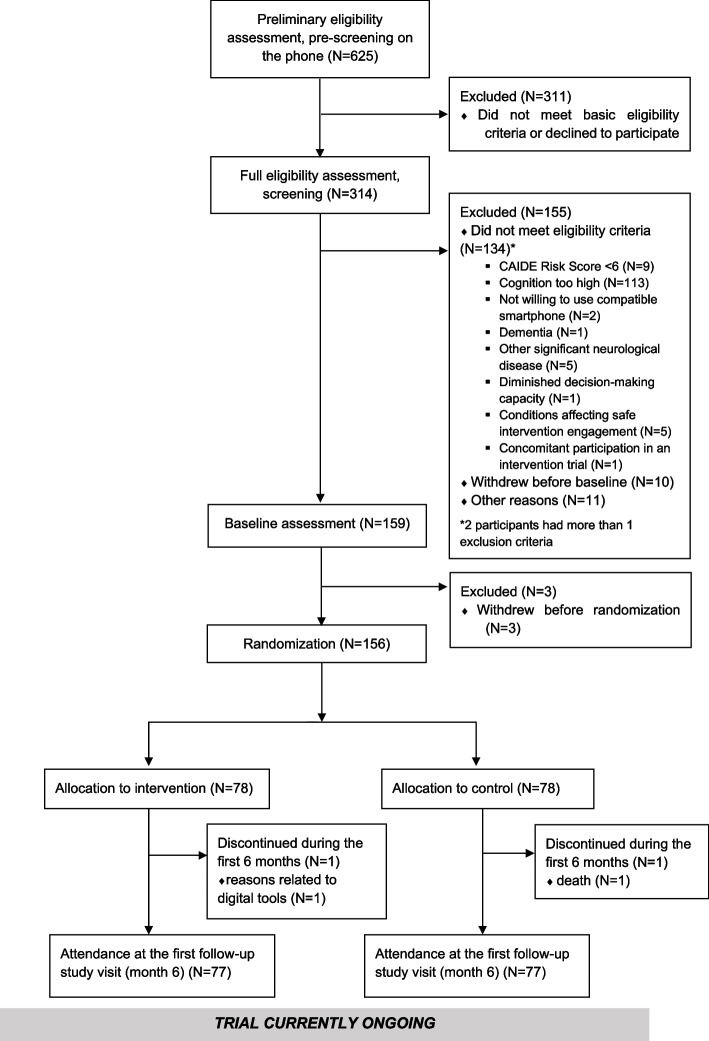
Study participant flow diagram (data until the first post-baseline study visit at month 6)

Recruitment started in September 2022 in Austria, November 2022 in Finland, and January 2023 in Sweden and Italy. In total, 625 individuals were pre-screened for a preliminary check of selected enrolment criteria, and 314 (50.24%) underwent the full screening to assess eligibility (Fig. 3). Most common reason for ineligibility was having cognitive performance above the inclusion criteria. Baseline assessment was conducted for 159 individuals (50.5% of screened individuals), and 156 were randomized. First participant was randomized in February 2023, and last one in June 2023. Trial is ongoing and will be completed by summer 2025. Retention until the first post-baseline visit at 6 months is high (*n* = 2 discontinued, retention 98.7%; Fig. 3).

Selected participant characteristics are shown in Table 5. Recruitment is balanced across the sites. At baseline, participants were on average 68.8 (SD 4.5) years old, had 14.9 (SD 3.1) years of education, and 64.7% were women. Cognitive and functional capacity were well preserved: the median MMSE score was 28 (IQR 27–29), CDR-SB 0 (IQR 0–0.5), and IADL 8 (IQR 8–8). The median CAIDE score was 8 (IQR 7–9). Vascular/lifestyle risk factors were common: 64.7% had a diagnosed hypertension (elevated systolic and diastolic BP were measured in 39.1% and 16.0% of the participants, respectively), 70.5% were overweight, 69.2% had hypercholesterolemia, 13.5% had diabetes, and 39.1% were physically inactive. Participant characteristics did not differ between the groups, apart from sex (more women in the control than in the intervention group, 73.1% vs. 56.4%, *p* = 0.03) and elevated systolic BP (more often in the intervention group than in the control group, 47.4% vs. 30.8%, *p* = 0.03). Participants’ smartphone and Internet usage prior to the trial is shown in Table 6. The majority used their phone frequently throughout the day (84% used it at least 6 times per day), and the most common purposes were messaging/chatting (96%) and phone calls (93%). Two-thirds of the participants had prior experience with using their smartphone to track health; approximately 48% had used lifestyle-related apps.

**Table 5:** Selected baseline characteristics of randomized participants

	**All** ***N* = 156**	**Intervention** ***N* = 78**	**Control** ***N* = 78**	***P*-value**
Study site				
Austria	40 (25.6%)	20 (25.6%)	20 (25.6%)	
Finland	40 (25.6%)	20 (25.6%)	20 (25.6%)	
Italy	40 (25.6%)	20 (25.6%)	20 (25.6%)	
Sweden	36 (23.1%)	18 (23.1%)	18 (23.1%)	
*Demographics*				
Age, years	68.8 (4.5), range 60–77	68.6 (4.5), range 60–77	69.0 (4.5), range 60–77	0.66
Sex, women	101 (64.7%)	44 (56.4%)	57 (73.1%)	**0.03**
Education, years	14.9 (3.1)	14.8 (3.0)	15.0 (3.2)	0.61
*Cognition, functional performance, dementia risk*				
MMSE	28 [27–29]	28 [27–29]	28 [28–29]	0.98
CDR-SB	0 [0–0.5]	0 [0–0.5]	0 [0–0.5]	0.34
IADL	8 [8–8]	8 [8–8]	8 [8–8]	0.68
CAIDE score	8 [7–9]	8 [7–9]	9 [7–10]	0.29
Self-reported concern over own cognition	65 (41.7%)	29 (37.2%)	36 (46.2%)	0.26
*Vascular/metabolic factors*				
SBP, mmHg	137.8 (17.5)	140.0 (18.1)	135.6 (16.7)	0.12
SBP > 140 mmHg	61 (39.1%)	37 (47.4%)	24 (30.8%)	**0.03**
DBP, mmHg	81.9 (9.4)	82.2 (9.1)	81.6 (9.7)	0.71
DBP > 90 mmHg	25 (16.0%)	12 (15.4%)	13 (16.7%)	0.83
BMI, kg/m2	27.7 (4.7)	27.6 (4.4)	27.7 (4.9)	0.86
BMI > 25 kg/m2	110 (70.5%)	55 (70.5%)	55 (70.5%)	1.00
Total cholesterol, mmol/l	5.3 (1.2)	5.3 (1.2)	5.3 (1.3)	0.77
LDL, mmol/l	3.1 (1.1)	3.0 (1.0)	3.1 (1.2)	0.80
HDL, mmol/l	1.7 (0.4)	1.6 (0.4)	1.7 (0.4)	0.51
Fasting glucose, mmol/l	5.7 (0.8)	5.8 (0.9)	5.7 (0.8)	0.50
Physical activity less frequently than twice/week	61 (39.1%)	29 (37.2%)	32 (41.0%)	0.62
*Cardiovascular and metabolic diseases*				
Hypertension	101 (64.7%)	52 (66.7%)	49 (62.8%)	0.62
Hypercholesterolemia	108 (69.2%)	51 (65.4%)	57 (73.1%)	0.30
Diabetes (type 1 or 2)	21 (13.5%)	13 (16.7%)	8 (10.3%)	0.24

Data are shown as N (%) for categorical variables, and as mean (standard deviation) or median [interquartile range] for continuous variables. CDR-SB is missing for *N* = 8 and fasting glucose for *N* = 1. Group comparisons were conducted using the chi-square test, Student’s t-test, and Mann–Whitney U test

*Abbreviations*: *BMI* body mass index, *CAIDE* Cardiovascular Risk Factors, Aging and Dementia, *CDR-SB* Clinical Dementia Rating Sum of Boxes, *DBP* diastolic blood pressure, *HDL* high-density lipoprotein, *IADL* Instrumental Activities of Daily Living, *LDL* low-density lipoprotein, *MMSE* Mini-Mental State Examination, *SBP* systolic blood pressure

**Table 6:** Participants’ smartphone and Internet usage at baseline

Question	N (% of respondents)
1. **How often do you use your smartphone during the day?**	
> 10 times	94 (60.3%)
6–10 times	37 (23.7%)
2–5 times	18 (11.5%)
< 2 times	0 (0.0%)
I cannot say	7 (4.5%)
2. **For which purposes do you use your smartphone? Multiple answers possible**	
Instant messaging/chatting e.g., WhatsApp	149 (95.5%)
Phone calls	145 (92.9%)
Taking photos/videos or looking at photos/watching videos	123 (78.8%)
Entertainment, e.g., playing games or social media e.g., Facebook)	111 (71.2%)
Organization (e.g., taking notes or using the calendar)	93 (59.6%)
Other	19 (12.2%)
3. **Have you already used your phone for tracking health? Multiple answers possible**	
Yes: I’ve used not health-related functions of my phone (e.g., setting reminders for medication intake, entering doctor appointments in the calendar)	61 (39.6%)
Yes: I’ve used apps supporting a healthy lifestyle (e.g., fitness or diet apps)	74 (48.1%)
Yes: I’ve used apps designed to help in the management of chronic diseases (e.g., a digital blood pressure diary)	13 (8.4%)
Yes: other	1 (0.6%)
No	51 (33.1%)
4. **How often do you use/access the Internet?**	
Several times a day every day	147 (94.8%)
Once a day	5 (3.2%)
2–6 times per week	3 (1.9%)
Once a week or less frequently	0 (0.0%)
5. **Which device do you regularly use to access the Internet? Multiple answers possible**	
Smartphone	152 (97.4%)
Computer/Laptop	114 (73.1%)
Tablet	62 (39.7%)
Other (including Smart TV or smartwatch)	42 (26.9%)
6. **For which purposes do you usually access the Internet? Multiple answers possible**	
Finding information	148 (95.5%)
Sending emails	137 (88.4%)
Reading news	131 (84.5%)
Paying bills/online banking	124 (80.0%)
eHealth (e.g., booking doctor appointments, checking test results)	97 (62.6%)
Social networks (e.g., Facebook, Twitter, Instagram)	87 (56.1%)
Bookings (e.g., holidays, transportation, theatre/concert tickets, restaurant)	84 (54.2%)
Online shopping	81 (52.3%)
Watching movies, TV, videos, listening to music	51 (32.9%)
Playing games	37 (23.9%)
Other	2 (1.3%)

Data available for all participants (*N* = 156), except for Q3 (*N* = 154), Q4 (*N* = 155), and Q6 (*N* = 155)

First results of the app usage during the first 6 months indicate that among the participants in the intervention group using the full LETHE App, mean of 50.5% (SD 7.5) used it daily. In the control group, 27.5% (SD 6.7) used their simplified version of the app daily (*p* < 0.001 for the difference between intervention and control groups). The median duration of a single session of app usage was 42.1 s (IQR 15.6–115.0) in the intervention group and 30.9 s (IQR 11.7–94.7) in the control group (*p* < 0.001). A digital cognitive testing was conducted 1 month after baseline visit, and altogether 153 participants out of 155 participants still in the trial completed the test.

## Discussion

The multinational LETHE RCT tests the feasibility of a digitally supported FINGER-based multimodal lifestyle intervention to promote brain health and reduce the risk of cognitive decline among at-risk older adults. The benefits of FINGER have been demonstrated in terms of cognition [6], dementia risk reduction [8, 9], daily functioning [44], quality of life [45], and several other outcomes [7, 46]. Complementing FINGER with digital elements can streamline the intervention delivery, support risk factor self-management, and enable the collection of novel digital biomarkers [16, 17]. Combined with other comprehensive data collected in this RCT (cognitive, clinical, lifestyle, and AD/dementia blood and imaging biomarkers), digital markers could help capture early disease-driven changes not detectable by standard methods, and identify the most relevant and actionable risk factors for different individuals (in line with the precision prevention concept [47]).

The adoption of digital tools and services among older adults is increasing, and the COVID-19 pandemic accelerated this change [48]. In the context of chronic disease prevention, digital/remote interventions are attractive as they could offer a cost-effective solution for risk factor self-management, with minimal burden to health care systems. In recent years, online/digital programs for cardiovascular risk improvement [49] and type 2 diabetes prevention [50] have been followed by brain health programs aimed at ameliorating risk factors relevant for dementia [51, 52]. Such programs have potential to induce benefits, yet evidence remains inconclusive due to the heterogeneity of the studies and interventions, and scarcity of controlled trial designs and longer-term (> 1 year) follow-ups [51, 52]. The available programs (especially apps) have often also not been rigorously assessed in high-quality studies, nor are they designed specifically for older adults with their needs and preferences in mind [43]. LETHE addresses these gaps by investigating an exceptionally long (two-year), digitally supported multimodal lifestyle intervention (aligned with original FINGER), both in terms of feasibility and impact on dementia risk. Feasibility assessment includes also qualitative evaluation of participant experiences, which is essential to identify the key elements of scalable digital solutions with optimal acceptance in the older population.

The LETHE intervention follows an innovative hybrid model, i.e., the individual and group-based activities (face-to-face and remote) are combined with independent digital activities. Another key element in this model are the digital coaches who have a crucial role in onboarding participants and providing technical assistance throughout the study, also in person. The in-person contact helps deliver the intervention with sufficient intensity and progression, support engagement with the technology, establish a trustworthy relationship with professionals, and strengthen social interaction among participants. Real-life human support, both peer and professional, is known to be important for older adults’ motivation and engagement in lifestyle and brain health interventions [13, 32, 53, 54]. Active engagement is crucial because it correlates closely with beneficial intervention effects on cognition and vascular risk profile [12, 15]. To our knowledge, the other ongoing or recently completed online/mHealth-based dementia risk reduction interventions are fully digital/remote even if certain coaching elements may be included [55–60], and they have faced challenges with recruitment, adherence, and retention. For instance, in the three-year Australian Maintain Your Brain RCT where an online multimodal intervention yielded cognitive benefits [56, 61], only about 6% of all invited older adults were enrolled (those with more risk factors and hence better risk reduction potential were less likely to participate) [62], and drop-out rate was high already after the first year [63]. A similar low recruitment rate was observed in the 18-month Healthy Aging Through Internet Counselling in the Elderly RCT where the first steps were taken to build an online lifestyle and vascular risk factor management platform for European older adults [60, 64]. These findings together with our first results from LETHE (high retention until six months, positive participant feedback, relatively high proportion of participants using app daily) support the concept of a hybrid multimodal intervention.

In line with FINGER, LETHE targets older adults at risk of dementia but without substantial cognitive impairment. The baseline data indicate that the desired target group with risk reduction potential was indeed successfully recruited (e.g., large proportion of participants with several modifiable risk factors). Our primary recruitment strategy (social media) is a fairly new approach in this target group, and it was chosen to reach persons in the right geographical area and age range, and with digital skills and interest. This strategy was efficient: e.g., > 1000 persons left their contact details within a few days in Finland and Sweden, respectively. Participants in LETHE are slightly younger than e.g., in FINGER, and predominantly female, which is common in online dementia prevention RCTs [52, 62]. The population is also highly educated, again in line with previous studies [62], and participants were regular users of smart devices and Internet prior to enrolment as expected based on the inclusion criteria. As a pilot study, LETHE will provide important information on how to develop the digital FINGER intervention concept; however, efforts must be made in the future to also reach and understand the needs of older adults with lower socioeconomic status and those less familiar with technology. Ongoing studies such as e.g., PRODEMOS [55] can be informative in this regard. Another key consideration is geographical and cultural context. A strength of LETHE is the involvement of European countries with varying digital readiness (e.g., Internet used regularly in 2022 by 54% of older adults in Italy, 64% in Austria, and > 80% in Finland and Sweden [18]). LETHE will thus also inform the wider research community and the World-Wide FINGERS network, where researchers from more than 60 countries are currently testing and adapting the FINGER model in diverse settings [65].

## Conclusions

The two-year LETHE pilot feasibility RCT combines in an innovative way the FINGER multimodal lifestyle intervention concept with technology, allowing a more personalized approach. LETHE explores a range of digital solutions (smartphone, mobile app, smartwatch, and novel interactive technology), which will provide much needed information on how older adults perceive the user-friendliness of technology and its meaningfulness in the context of brain health and dementia risk reduction. The hybrid intervention design combining in-person sessions and independent digital activities will inform future studies about the optimal frequency and balance between digital components and human support. The comprehensive data collection (also including AD/dementia biomarkers and passive digital biomarkers) is unique for digital RCTs in this target population and can give rise to new hypotheses of the mechanisms and early changes preceding cognitive decline.

## Supplementary Information

Supplementary Material 1.

## Data Availability

The LETHE consortium is open to requests from external researchers for data collected in this study. Applicants will be asked to submit a study protocol, including the research question, planned analysis, and data required. Data controllers will evaluate this plan (i.e., relevance of the research question, suitability of data, quality of proposed analyses, planned/ongoing LETHE analyses, and other matters) on a case-by-case basis and provide the data or reject the request. Shared data will encompass the data dictionary and de-identified data only. Any analysis will be conducted in collaboration with the LETHE Group. Access is subject to the LETHE legal framework. An access agreement will be prepared and signed by relevant parties. Applications for data will be considered after the trial results have been published and data will be made available according to the terms of the access agreement.

## Abbreviations

AB: Advisory board
AD: Alzheimer’s disease
BMI: Body mass index
BP: Blood pressure
CAIDE: Cardiovascular Risk Factors, Aging, and Incidence of Dementia
CDR-SB: Clinical Dementia Rating – Sum of Boxes
CTMS: Clinical Trial Management System
cTRAIN: Cognitive training program used in LETHE
FINGER: Finnish Geriatric Intervention Study to Prevent Cognitive Impairment and Disability
IADL: Instrumental Activities of Daily Living
LIBRA: LIfestyle for BRAin health
MMSE: Mini-Mental State Examination
NTB: Neuropsychological Test Battery
RCT: Randomized controlled trial

## References

[1] World Health Organization. Risk reduction of cognitive decline and dementia: WHO guidelines. Geneva. 2019. Available from: https://www.who.int/publications/i/item/9789241550543. Accessed 21 Nov 2023. 31219687

[2] Long S, Benoist C, Weidner W. World Alzheimer Report 2023: Reducing dementia risk: never too early, never too late. London: England; 2023.

[3] Livingston G, Huntley J, Sommerlad A, Ames D, Ballard C, Banerjee S, et al. Dementia prevention, intervention, and care: 2020 report of the Lancet Commission. The Lancet. 2020;396(10248):413–46. 10.1016/S0140-6736(20)30367-6 PMC7392084 32738937

[4] Kivipelto M, Mangialasche F, Ngandu T. Lifestyle interventions to prevent cognitive impairment, dementia and Alzheimer disease. Nat Rev Neurol. 2018;14(11):653–66. 30291317 10.1038/s41582-018-0070-3

[5] Barbera M, Perera D, Matton A, Mangialasche F, Rosenberg A, Middleton L, et al. Multimodal Precision Prevention - A New Direction in Alzheimer’s Disease. J Prevent Alzheimer’s Dis. 2023;10(4):718–28. 10.14283/jpad.2023.114 37874092

[6] Ngandu T, Lehtisalo J, Solomon A, Levälahti E, Ahtiluoto S, Antikainen R, et al. A 2 year multidomain intervention of diet, exercise, cognitive training, and vascular risk monitoring versus control to prevent cognitive decline in at-risk elderly people (FINGER): A randomised controlled trial. The Lancet. 2015;385(9984):2255–63. 10.1016/S0140-6736(15)60461-5 25771249

[7] Lehtisalo J, Rusanen M, Solomon A, Antikainen R, Laatikainen T, Peltonen M, et al. Effect of a multi-domain lifestyle intervention on cardiovascular risk in older people: the FINGER trial. Eur Heart J. 2022;43(21):2054–61. 35051281 10.1093/eurheartj/ehab922 PMC9156384

[8] Solomon A, Handels R, Wimo A, Antikainen R, Laatikainen T, Levälahti E, et al. Effect of a multidomain lifestyle intervention on estimated dementia risk. J Alzheimer’s Dis. 2021;82(4):1461–6. 34151805 10.3233/JAD-210331 PMC8461663

[9] Deckers K, Köhler S, Ngandu T, Antikainen R, Laatikainen T, Soininen H, et al. Quantifying dementia prevention potential in the FINGER randomized controlled trial using the LIBRA prevention index. Alzheimer’s and Dementia. 2021;17(7):1205–12. 33403822 10.1002/alz.12281 PMC8359273

[10] Kulmala J, Ngandu T, Kivipelto M. Prevention Matters: Time for Global Action and Effective Implementation. J Alzheimer’s Dis. 2018;64(s1):S191–8. 29504541 10.3233/JAD-179919

[11] Coley N, Ngandu T, Lehtisalo J, Soininen H, Vellas B, Richard E, et al. Adherence to multidomain interventions for dementia prevention: Data from the FINGER and MAPT trials. Alzheimer’s Dementia. 2019;15(6):729–41. 31047857 10.1016/j.jalz.2019.03.005

[12] Ngandu T, Lehtisalo J, Korkki S, Solomon A, Coley N, Antikainen R, et al. The effect of adherence on cognition in a multidomain lifestyle intervention (FINGER). Alzheimer’s Dementia. 2022;18(7):1325–34. 34668644 10.1002/alz.12492

[13] Coley N, Coniasse-Brioude D, Igier V, Fournier T, Poulain JP, Andrieu S, et al. Disparities in the participation and adherence of older adults in lifestyle-based multidomain dementia prevention and the motivational role of perceived disease risk and intervention benefits: an observational ancillary study to a randomised controlled trial. Alzheimers Res Ther. 2021;13(1):157. 10.1186/s13195-021-00904-6 PMC8464095 34560903

[14] Beishuizen CRL, Coley N, Moll van Charante EP, van Gool WA, Richard E, Andrieu S. Determinants of Dropout and Nonadherence in a Dementia Prevention Randomized Controlled Trial: The Prevention of Dementia by Intensive Vascular Care Trial. J Am Geriatr Soc. 2017;65(7):1505–13. 28263374 10.1111/jgs.14834

[15] Coley N, Andre L, Hoevenaar-Blom MP, Ngandu T, Beishuizen C, Barbera M, et al. Factors Predicting Engagement of Older Adults With a Coach-Supported eHealth Intervention Promoting Lifestyle Change and Associations Between Engagement and Changes in Cardiovascular and Dementia Risk: Secondary Analysis of an 18-Month Multinational Randomized Controlled Trial. J Med Internet Res. 2022;24(5):e32006. 10.2196/32006 PMC9127655 35385395

[16] Kourtis LC, Regele OB, Wright JM, Jones GB. Digital biomarkers for Alzheimer’s disease: the mobile/wearable devices opportunity. NPJ Digit Med. 2019;2:9. 10.1038/s41746-019-0084-2 PMC6526279 31119198

[17] Brem AK, Kuruppu S, de Boer C, Muurling M, Diaz-Ponce A, Gove D, et al. Digital endpoints in clinical trials of Alzheimer’s disease and other neurodegenerative diseases: challenges and opportunities. Front Neurol. 2023;14:1210974. 10.3389/fneur.2023.1210974 PMC10332162 37435159

[18] EU Eurostat. Digital Economy and Society Database. https://ec.europa.eu/eurostat/web/digital-economy-and-society/database. Accessed 21 Nov 2023.

[19] LETHE Consortium. LETHE project homepage. https://www.lethe-project.eu/. Accessed 21 Nov 2023.

[20] Hanke S, Mangialasche F, Bödenler M, Neumayer B, Ngandu T, Mecocci P, et al. AI-Based Predictive Modelling of the Onset and Progression of Dementia. Smart Cities. 2022;5(2):700–14.

[21] Kivipelto M, Ngandu T, Laatikainen T, Winblad B, Soininen H, Tuomilehto J. Risk score for the prediction of dementia risk in 20 years among middle aged people: a longitudinal, population-based study. Lancet Neurol. 2006;5(9):735–41. 16914401 10.1016/S1474-4422(06)70537-3

[22] Holden RJ. A Simplified System Usability Scale (SUS) for Cognitively Impaired and Older Adults. Proceed Int Symp Human Fact Ergon Health Care. 2020;9(1):180–2.

[23] Schiepers OJG, Köhler S, Deckers K, Irving K, O’Donnell CA, van den Akker M, et al. Lifestyle for Brain Health (LIBRA): a new model for dementia prevention. Int J Geriatr Psychiatry. 2018;33(1):167–75. 28247500 10.1002/gps.4700

[24] Exalto LG, Quesenberry CP, Barnes D, Kivipelto M, Biessels GJ, Whitmer RA. Midlife risk score for the prediction of dementia four decades later. Alzheimer’s & Dementia. 2014;10(5):562–70. 10.1016/j.jalz.2013.05.1772 24035147

[25] Vos SJB, Van Boxtel MPJ, Schiepers OJG, Deckers K, De Vugt M, Carrière I, et al. Modifiable Risk Factors for Prevention of Dementia in Midlife, Late Life and the Oldest-Old: Validation of the LIBRA Index. J Alzheimer’s Dis. 2017;58(2):537–47. 28453475 10.3233/JAD-161208

[26] Harrison J, Psychol C, Minassian SL, Jenkins L, Black RS, Koller M, et al. A Neuropsychological Test Battery for Use in Alzheimer Disease Clinical Trials. Arch Neurol. 2007;64(9):1323–9. 17846273 10.1001/archneur.64.9.1323

[27] Barbera M, Lehtisalo J, Perera D, Aspö M, Cross M, De Jager Loots CA, et al. A multimodal precision-prevention approach combining lifestyle intervention with metformin repurposing to prevent cognitive impairment and disability: the MET-FINGER randomised controlled trial protocol. Alzheimers Res Ther. 2024;16(1):23. 38297399 10.1186/s13195-023-01355-x PMC10829308

[28] Sindi S, Thunborg C, Rosenberg A, Andersen P, Andrieu S, Broersen LM, et al. Multimodal Preventive Trial for Alzheimer’s Disease: MIND-ADmini Pilot Trial Study Design and Progress. J Prevent Alzheimer’s Dis. 2022;9(1):30–9. 10.14283/jpad.2022.4 PMC8783958 35098971

[29] Lehtisalo J, Ngandu T, Valve P, Antikainen R, Laatikainen T, Strandberg T, et al. Nutrient intake and dietary changes during a 2-year multi-domain lifestyle intervention among older adults: Secondary analysis of the Finnish Geriatric Intervention Study to Prevent Cognitive Impairment and Disability (FINGER) randomised controlled trial. Br J Nutr. 2017;118(4):291–302. 28875868 10.1017/S0007114517001982

[30] World Health Organization. WHO guidelines on physical activity and sedentary behaviour. Geneva. 2020. https://www.who.int/publications/i/item/9789240015128. Accessed 21 Nov 2023. 33369898

[31] Turunen M, Hokkanen L, Bäckman L, Stigsdotter-Neely A, Hänninen T, Paajanen T, et al. Computer-based cognitive training for older adults: Determinants of adherence. PLoS One. 2019;14(7):e0219541. 10.1371/journal.pone.0219541 PMC6620011 31291337

[32] Akenine U, Thunborg C, Kivipelto M, Fallahpour M. Experiences of Participation in a Multimodal Preventive Trial MIND-ADMINI Among Persons with Prodromal Alzheimer’s Disease: A Qualitative Study. J Multidiscip Healthc. 2022;15:219–34. 35125872 10.2147/JMDH.S345607 PMC8811792

[33] Luo J, Beam CR, Gatz M. Is Stress an Overlooked Risk Factor for Dementia? A Systematic Review from a Lifespan Developmental Perspective. Prev Sci. 2023;24(5):936–49. 35622193 10.1007/s11121-022-01385-1

[34] Shi L, Chen SJ, Ma MY, Bao YP, Han Y, Wang YM, et al. Sleep disturbances increase the risk of dementia: A systematic review and meta-analysis. Sleep Med Rev. 2018;40:4–16. 28890168 10.1016/j.smrv.2017.06.010

[35] Fogg B, Hreha J. Behavior Wizard: A Method for Matching Target Behaviors with Solutions. In: Ploug T, Hasle P, Oinas-Kukkonen H, editors. Persuasive Technology. PERSUASIVE 2010. Lecture Notes in Computer Science, vol 6137. Heidelberg: Springer; 2010. p. 117–131.

[36] Wood W, Neal DT. Healthy through habit: Interventions for initiating & maintaining health behavior change. Behav Sci Policy. 2016;2(1):71–83.

[37] Harjumaa M, Absetz P, Ermes M, Mattila E, Männikkö R, Tilles-Tirkkonen T, et al. Internet-Based lifestyle intervention to prevent type 2 diabetes through healthy habits: Design and 6-Month usage results of randomized controlled trial. JMIR Diabetes. 2020;5(3):e15219. 10.2196/15219 PMC7448183 32779571

[38] Stop Diabetes Consortium. Tiny Habits library. https://sites.uef.fi/stopdia/t2d-data-pienet-teot/. Accessed 21 Nov 2023.

[39] Thabane L, Hopewell S, Lancaster GA, Bond CM, Coleman CL, Campbell MJ, et al. Methods and processes for development of a CONSORT extension for reporting pilot randomized controlled trials. Pilot Feasibility Stud. 2016;2:25. 10.1186/s40814-016-0065-z PMC5153862 27965844

[40] Forcano L, Fauria K, Soldevila-Domenech N, Minguillón C, Lorenzo T, Cuenca-Royo A, et al. Prevention of cognitive decline in subjective cognitive decline APOE ε4 carriers after EGCG and a multimodal intervention (PENSA): Study design. Alzheimer’s and Dementia: Translational Research and Clinical Interventions. 2021;7(1):e12155. 10.1002/trc2.12155 PMC8012239 33816762

[41] Chew KA, Xu X, Siongco P, Villaraza S, Phua AKS, Wong ZX, et al. SINgapore GERiatric intervention study to reduce physical frailty and cognitive decline (SINGER)–pilot: A feasibility study. Alzheimer’s and Dementia: Translational Research and Clinical Interventions. 2021;7(1):e12141. 10.1002/trc2.12141 PMC7958306 33748399

[42] Park HK, Jeong JH, Moon SY, Park YK, Hong CH, Na HR, et al. South Korean study to prevent cognitive impairment and protect brain health through lifestyle intervention in at-risk elderly people: Protocol of a multicenter, randomized controlled feasibility trial. J Clin Neurol (Korea). 2020;16(2):292–303. 10.3988/jcn.2020.16.2.292 PMC7174118 32319247

[43] Andre L, Giulioli C, Piau A, Bongard V, Richard E, Moll van Charante EP, et al. Telephone and Smartphone-Based Interventions for Cognitive and Cardio-Metabolic Health in Middle-Aged and Older Adults: A Systematic Review. Clin Interv Aging. 2022;17:1599–624. 36393902 10.2147/CIA.S352137 PMC9661915

[44] Kulmala J, Ngandu T, Havulinna S, Levälahti E, Lehtisalo J, Solomon A, et al. The Effect of Multidomain Lifestyle Intervention on Daily Functioning in Older People. J Am Geriatr Soc. 2019;67(6):1138–44. 30809801 10.1111/jgs.15837

[45] Strandberg TE, Levälahti E, Ngandu T, Solomon A, Kivipelto M, Kivipelto M, et al. Health-related quality of life in a multidomain intervention trial to prevent cognitive decline (FINGER). Eur Geriatr Med. 2017;8(2):164–7.

[46] Marengoni A, Rizzuto D, Fratiglioni L, Antikainen R, Laatikainen T, Lehtisalo J, et al. The Effect of a 2-Year Intervention Consisting of Diet, Physical Exercise, Cognitive Training, and Monitoring of Vascular Risk on Chronic Morbidity-the FINGER Randomized Controlled Trial. J Am Med Dir Assoc. 2018;19(4):355–360.e1. 29108888 10.1016/j.jamda.2017.09.020

[47] Frisoni GB, Molinuevo JL, Altomare D, Carrera E, Barkhof F, Berkhof J, et al. Precision prevention of Alzheimer’s and other dementias: Anticipating future needs in the control of risk factors and implementation of disease-modifying therapies. Alzheimer’s and Dementia. 2020;16(10):1457–68. 32815289 10.1002/alz.12132

[48] Lehtisalo J, Palmer K, Mangialasche F, Solomon A, Kivipelto M, Ngandu T. Changes in Lifestyle, Behaviors, and Risk Factors for Cognitive Impairment in Older Persons During the First Wave of the Coronavirus Disease 2019 Pandemic in Finland: Results From the FINGER Study. Front Psychiatry. 2021;12:624125. 10.3389/fpsyt.2021.624125 PMC7907420 33643095

[49] Beishuizen CRL, Stephan BCM, Van Gool WA, Brayne C, Peters RJG, Andrieu S, et al. Web-based interventions targeting cardiovascular risk factors in middle-aged and older people: A systematic review and meta-analysis. J Med Internet Res. 2016;18(3):e55. 10.2196/jmir.5218 PMC4808240 26968879

[50] Van Rhoon L, Byrne M, Morrissey E, Murphy J, McSharry J. A systematic review of the behaviour change techniques and digital features in technology-driven type 2 diabetes prevention interventions. Digit Health. 2020;6:2055207620914427. 10.1177/2055207620914427 PMC7093696 32269830

[51] Wesselman LMP, Hooghiemstra AM, Schoonmade LJ, De Wit MCJ, Van Der Flier WM, Sikkes SAM. Web-based multidomain lifestyle programs for brain health: Comprehensive overview and meta-analysis. JMIR Ment Health. 2019;6(4):e12104. 10.2196/12104 PMC6477576 30964438

[52] Whitfield T, McConnell B, Renouf P, Mansour H, Zabihi S, Aguirre E, et al. The effect of remotely delivered lifestyle interventions on cognition in older adults without dementia: A systematic review and meta-analysis. Ageing Res Rev. 2021;72:101505. 10.1016/j.arr.2021.101505 PMC9761296 34757173

[53] Zhang J, Eggink E, Zhang X, Li X, Jiang B, Liu H, et al. Needs and views on healthy lifestyles for the prevention of dementia and the potential role for mobile health (mHealth) interventions in China: a qualitative study. BMJ Open. 2022;12(11):e061111. 10.1136/bmjopen-2022-061111 PMC9684993 36414280

[54] Coley N, Rosenberg A, van Middelaar T, Soulier A, Barbera M, Guillemont J, et al. Older Adults’ Reasons for Participating in an eHealth Prevention Trial: A Cross-Country, Mixed-Methods Comparison. J Am Med Dir Assoc. 2019;20(7):843–849.e5. 30541689 10.1016/j.jamda.2018.10.019

[55] Eggink E, Hafdi M, Hoevenaar-Blom MP, Song M, Andrieu S, Barnes LE, et al. Prevention of dementia using mobile phone applications (PRODEMOS): Protocol for an international randomised controlled trial. BMJ Open. 2021;11(6):e049762. 10.1136/bmjopen-2021-049762 PMC8191602 34108173

[56] Heffernan M, Andrews G, Fiatarone Singh MA, Valenzuela M, Anstey KJ, Maeder AJ, et al. Maintain Your Brain: Protocol of a 3-Year Randomized Controlled Trial of a Personalized Multi-Modal Digital Health Intervention to Prevent Cognitive Decline among Community Dwelling 55 to 77 Year Olds. J Alzheimer’s Dis. 2019;70(s1):S221–37. 30475762 10.3233/JAD-180572 PMC6700632

[57] Gray M, Madero EN, Gills JL, Paulson S, Jones MD, Campitelli A, et al. Intervention for a Digital, Cognitive, Multi-Domain Alzheimer Risk Velocity Study: Protocol for a Randomized Controlled Trial. JMIR Res Protoc. 2022;11(2):e31841. 35119374 10.2196/31841 PMC8857690

[58] Amos JG, Zheng L, Eramudugolla R, Parekh D, Huque MH, Delbaere K, et al. MyCOACH (COnnected Advice for Cognitive Health): a digitally delivered multidomain intervention for cognitive decline and risk of dementia in adults with mild cognitive impairment or subjective cognitive decline-study protocol for a randomised controlled trial. BMJ Open. 2023;13(10):e075015. 37903606 10.1136/bmjopen-2023-075015 PMC10619101

[59] Feldman HH, Belleville S, Nygaard HB, Montero-Odasso M, Durant J, Lupo JL, et al. Protocol for the Brain Health Support Program Study of the Canadian Therapeutic Platform Trial for Multidomain Interventions to Prevent Dementia (CAN-THUMBS UP): A Prospective 12-Month Intervention Study. J Prevent Alzheimer’s Dis. 2023;10(4):875–85. 10.14283/jpad.2023.65 PMC10258470 37874110

[60] Richard E, Moll van Charante EP, Hoevenaar-Blom MP, Coley N, Barbera M, van derGroep A, et al. Healthy ageing through internet counselling in the elderly (HATICE): a multinational, randomised controlled trial. Lancet Digit Health. 2019;1(8):e424–34. 33323224 10.1016/S2589-7500(19)30153-0

[61] Brodaty H, Valenzuela M, Singh MAF, Sachdev PS, McNeil J, Lautenschlager NT, et al. Maintain Your Brain: outcomes of an online program to prevent cognitive decline with aging. Alzheimer’s & Dementia. 2023;19(S22):e075183.

[62] Welberry HJ, Chau T, Heffernan M, San Jose JC, Jorm LR, Singh MF, et al. Factors Associated with Participation in a Multidomain Web-Based Dementia Prevention Trial: Evidence from Maintain Your Brain (MYB). J Alzheimer’s Dis. 2023;92(3):959–74. 36806506 10.3233/JAD-220990

[63] Brodaty H, Heffernan M, Anstey KJ, Fiatarone Singh MA, Jorm L, Lautenschlager NT, et al. Maintain Your Brain trial: Early findings and lessons learned from adherence and compliance data. Alzheimer’s & Dementia. 2021;17(S10):e053596.

[64] Barbera M, Mangialasche F, Jongstra S, Guillemont J, Ngandu T, Beishuizen C, et al. Designing an Internet-Based Multidomain Intervention for the Prevention of Cardiovascular Disease and Cognitive Impairment in Older Adults: The HATICE Trial. J Alzheimer’s Dis. 2018;62(2):649–63. 29480185 10.3233/JAD-170858

[65] Kivipelto M, Mangialasche F, Snyder HM, Allegri R, Andrieu S, Arai H, et al. World-Wide FINGERS Network: A global approach to risk reduction and prevention of dementia. Alzheimer’s Dementia. 2020;16(7):1078–94. 32627328 10.1002/alz.12123 PMC9527644

