# Supplementary material for: A digitally supported multimodal lifestyle program to promote brain health among older adults (the LETHE randomized controlled feasibility trial): study design, progress, and first results
Source: Alzheimers Res Ther. 2024 Nov 21;16:252. doi: 10.1186/s13195-024-01615-4 (PMC11580696; doi:10.1186/s13195-024-01615-4)
Supplement: Supplementary file 1 — Supplementary Material 1. [file 13195_2024_1615_MOESM1_ESM.pdf]

**ADDITIONAL FILE**

**Supplementary Table 1.** Key dietary intervention goals in nutrient and food intake level.

| **Nutrient-level goals** | **Food-level goals** |
|---|---|
| **Protein:** 15-20 E% | Limited consumption of red and processed meat (max 500 g/week) |
| **Fat:** Total fat 25-40 E%<br>Two main goals for quality:<br>1) Saturated fatty acids (SFA) ≤ 10 E%<br>2) Polyunsaturated fatty acids 5-10 E%<br>2/3 of total fat consists of polyunsaturated and monounsaturated fatty acids.<br>Omega-3 fatty acids 1 E% (DHA 200 mg/day) | Replacing butter and other SFA sources with vegetable fats.<br>Low-fat options in milk and meat products.<br>At least 2 portions of fatty fish a week. Fish oil supplements may be recommended for those not consuming fish (even after motivating to do so). |
| **Carbohydrates:** 45-60 E%<br>Two main goals for quality:<br>1) Refined sugar ≤ 10 E%<br>2) Dietary fibre 25-35 g/day | Limited intake of sugary beverages and foods with added sugar<br>Favouring whole grain products.<br>At least 200 g/day of fruit, berries<br>At least 200 g/day of vegetables |
| **Alcohol:** ≤ 5 E% | Max. 2 units/day (men) and 1 unit/day (women). Alcohol is not recommended for abstainers. |
| **Salt (NaCl):** ≤ 6 g/day | Favouring low-sodium options. |
| **Vitamins, supplements:**<br>Vitamin D supplement as per local guidelines. Current evidence does not support the use of other supplements to improve brain health (1); multivitamin/mineral supplements may be recommended for those judged to be at risk of deficiency. | |

Abbreviations: DHA = docosahexaenoic acid; E% = energy intake as % of total energy intake; SFA saturated fatty acids.

**Supplementary Table 2.** Progression of the strength training and aerobic exercise programs

| | Month 3 | Month 4–6 | Month 7–9 | Month 10–24 |
|---|---|---|---|---|
| *Resistance exercise* | | | | |
| Frequency/week | 1–2 | 1–2 | 2 | 2–3 |
| Duration (min) | 30–45 | 30–60 | 45–60 | 60 |
| Number of muscle groups | 8–10 | 8–10 | 8–10 | 8–10 |
| Repetitions/set | 8–15 | 8–20 | 8–20 | 8–20 |
| Load (% of estimated 1RM) | 40–50% | 60% | 70% | 70–80% |
| Sets | 2 | 2–3 | 1–3 | 2–3 |
| *Aerobic exercise* | | | | |
| Frequency/week | 1 | 2–3 | 3–4 | 3–5 |
| Duration (min) | 30–45 | 30–45 | 30–60 | 45–60 |

Abbreviations: RM = repetition maximum

The strength training sessions are designed to target the eight main muscle groups/exercises (knee extension and flexion, abdomen and back muscle exercises, rotation, upper back and arm muscle exercises, and leg press for lower extremity muscles). Elements of aerobic exercise can also be incorporated (e.g., circuit training). Sessions also include warm-up, cool-down/stretching, and balance training.

**Supplementary Table 3.** Short diet monitoring questionnaire in the LETHE App (for the intervention group)

| **Questions** | **Answers** | | | **Info** |
|---|---|---|---|---|
| | **0 points** | **1 point** | **2 points** | |
| **BLOCK 1** | | | | |
| In the past week, I've eaten fruits and berries | Less than one portion per day | 1 portion per day | 2 portions or more per day | One portion is e.g. one apple, one banana, or about 2 deciliter of fresh berries, or... [local examples added] |
| In the past week, I've eaten vegetables | Less than one portion per day | 1 portion per day | 2 portions or more per day | One portion is e.g. one carrot, 1 deciliter of sliced vegetables, or.. [local examples added; note to consider also cooked vegetables in meals] |
| In the past week, the bread and cereals I've eaten have been | Mostly "white" | Sometimes "white", sometimes wholegrain | Mostly wholegrain | White bread and cereal are e.g wheat bread, corn flakes, or [local examples added], whole grain options are e.g. oatmeal porrige, rye bread, [local examples added] |
| **BLOCK 2** | | | | |
| In the past week, I've eaten legumes (lentils, beans, chickpeas etc..) | Less than one portion per week | 1 portion per week | 2 portions or more per week | One portion is approx. 1 deciliter of boiled lentils or chickpeas or half a can of canned beans [local examples added]. Also consider bean-based protein products such as tofu.. [local examples added] |
| In the past week, I've eaten fish | Less than one portion per week | 1 portion per week | 2 portions or more per week | One portion is approx. 100-150 g baked or fried fish filet or one portion of fish soup [local examples added]. If you have eaten smaller amounts (e.g., gravlax on bread), consider the smaller portion when estimating total amount per week |
| In the past week, I've eaten red or processed meat | More than once per day | Once per day or almost once per day | 4-5 times a week or less | One portion approx. 100-150 g baked or fried meat, hamburger steak, or small sausage [local examples added] |

| | | | | |
|---|---|---|---|---|
| When cooking or dressing my food in the past week, I've used | Mostly fats of animal origin OR I do not pay attention | Both fats of animal and vegetable origin | Mostly or only fats of vegetable origin | Fats of vegetable origin include vegetable oils such as olive or rapeseed, or vegetable based margarin such as [local brand names added]. Fats of animal origin are butter and butter-based mixtures and lard |
| **BLOCK 3** | | | | |
| In the past week, I've consumed | 1-2 OR more than 6 meals & snack per day | 3-4 meals & snack per day | 5-6 meals & snack per day | A snack is an eating occasion when any energy-containing drink and/or food is eaten. Drinking only water is not considered a snack. |
| In the past week, I've chosen low-salt food (e.g. bread, spreads, ready meals) | Never OR I haven't paid attention | Sometimes | Always when available | Low-salt options are the ones marked with “heart” sign by the Heart Association or those with “low salt” mark on the package [local examples added]. You can also choose this option if you regularly compare the labels of the products and choose options lower in salt. |
| In the past week, I've eaten sweets and consumed sweetened drinks | Every day | 4-5 times per week | 2-3 times per week or less | Sweets are e.g. chocolate, candy, and confection [local examples added] and sugared drinks e.g. juice sweetened with sugar [local brand names added] or soft drinks [local brand names added]. |
| (for men) In the past week, I've drunk | More than 2 alcohol units per day | About 2 alcohol units per day | Less than 2 alcohol units per day | One portion of alcohol is e.g one small beer (3 deciliters), 12 cl of wine, or 4 cl strong spirit [locally available portion sizes adopted]. |
| (for women) In the past week, I've drunk | More than 1 alcohol unit per day | About 1 alcohol unit per day | Less than 1 alcohol unit per day | One portion of alcohol is e.g one small beer (3 deciliters), 12 cl of wine, or 4 cl strong spirit [locally available portion sizes adopted]. |

**Supplementary Table 4.** Examples of small practical everyday tips to improve lifestyle (tiny habits, included in the full LETHE App view for the intervention group)

| PLACE | TITLE | DESCRIPTION OF THE HABIT | HEALTH FACT |
|---|---|---|---|
| ***LIFESTYLE DOMAIN: DIET*** | | | |
| At home | A gentle start for the morning | I will have a smoothie for breakfast | I have no appetite early in the morning, but I can learn how to eat breakfast by choosing fresh, liquid foods, like a smoothie. Breakfast is important because it breaks the fast and gives me energy after the night. It will also stabilize my blood sugar levels and thus help increase alertness and control my eating during the rest of the day. |
| At the supermarket | Nuts giving energy while shopping | I will put nuts in my bag when I go shopping | In case I get hungry while shopping I will put nuts in my bag. A handful of nuts, almonds, or seeds contains unsaturated fats, dietary fiber, protein, and plenty of vitamins and minerals. Nuts will help me promote my heart health and control my weight. |
| ***LIFESTYLE DOMAIN: EXERCISE AND SITTING*** | | | |
| Out of home | Walk the dog | I will walk the dog today | Even a short walk counts as physical exertion and may enhance my wellbeing when done regularly. |
| Out of home | Steps while picking berries | I will go pick berries with my grandchildren | Wandering in the forest may improve my health and wellbeing in many ways. I get fitter and my stress levels are reduced. |
| ***LIFESTYLE DOMAIN: MENTAL WELLBEING*** | | | |
| At home | Return home relaxed | When I get home, I will relax by doing a quick breathing exercise. | Using relaxation techniques regularly may help me control stress and improve wellbeing. It may also alleviate symptoms of anxiety and depression and help with controlling high blood pressure, but it does not replace medical care. |
| At home | Have a nice day | Before I leave and close the front door, I wish my | By paying attention to my loved ones and being positive, I increase the wellbeing of both of us. |

| | | | |
|---|---|---|---|
| | | loved one a nice day | |
| ***LIFESTYLE DOMAIN: SLEEP*** | | | |
| At home | TV away from the bedroom | I move the TV out of the bedroom | Insomnia is primarily treated by self-management: take care of your sleep rhythm and make sure you have healthy lifestyle habits and appropriate sleeping conditions. If this does not help, short-term sleep medication may be needed. |
| At home | Blackout curtains help fall asleep | I use blackout curtains when the light interferes with sleep | Taking care of your sleeping conditions is one of the cornerstones of sleep self-management. Falling asleep in the dark is easier, and the curtains also ensure that the morning sun won't wake me up. |
| ***LIFESTYLE DOMAIN: COGNITIVE STIMULATION AND SOCIAL INTERACTION*** | | | |
| At home | Let's play | I'll solve a puzzle or play with legos with a (grand)child. | Comprehensive brain training in a nice company enhances its benefits and reduces stress. Social interaction itself trains different information processing skills. |
| At home | Learning by heart | I'll try to learn by heart and remember a password, door code, or a phone number. | Memorizing, storing, and retrieving things from memory activates and trains different memory domains. |
| ***LIFESTYLE DOMAIN: NON-SMOKING*** | | | |
| Anywhere | Support phone call and cigarette cravings | Whenever I feel like having a cigarette, I will call a loved one/friend | Cigarette craving is a bodily reaction to a decrease in nicotine levels. After smoking cessation, strong cravings can usually be experienced for a few weeks. They usually do not last more than 60-90 seconds, which means that the cravings will pass while we are talking on the phone. |
| Anywhere | Pleasure from music | I will listen to music that makes | Many smokers get pleasure from smoking, and quitting can initially cause feelings of depression which usually do not last longer than a few days. |

| | | | |
|---|---|---|---|
| | | me feel good for 10 minutes | It is important to think what else can bring joy, apart from cigarettes. Focusing on listening to my favorite music helps me cope without the pleasure I get from smoking cigarettes. |
